# Mutation spectrum in human colorectal cancers and potential functional relevance

**DOI:** 10.1186/1471-2350-14-32

**Published:** 2013-03-08

**Authors:** Hongzhuan Yin, Yichao Liang, Zhaopeng Yan, Baolin Liu, Qi Su

**Affiliations:** 1 Department of General Surgery, Shengjing Hospital of China Medical University, No. 36 Sanhao Street, Heping District, Shenyang, Liaoning Province 110004, China

**Keywords:** Colorectal cancers, Mutation spectrum, RNA-Seq, Transcriptome

## Abstract

**Background:**

Somatic variants, which occur in the genome of all cells, are well accepted to play a critical role in cancer development, as their accumulation in genes could affect cell proliferations and cell cycle.

**Methods:**

In order to understand the role of somatic mutations in human colorectal cancers, we characterized the mutation spectrum in two colorectal tumor tissues and their matched normal tissues, by analyzing deep-sequenced transcriptome data.

**Results:**

We found a higher mutation rate of somatic variants in tumor tissues in comparison with normal tissues, but no trend was observed for mutation properties. By applying a series of stringent filters, we identified 418 genes with tumor specific disruptive somatic variants. Of these genes, three genes in mucin protein family (*MUC2*, *MUC4*, and *MU12*) are of particular interests. It has been reported that the expression of mucin proteins was correlated with the progression of colorectal cancer therefore somatic variants within those genes can interrupt their normal expression and thus contribute to the tumorigenesis.

**Conclusions:**

Our findings provide evidence of the utility of RNA-Seq in mutation screening in cancer studies, and suggest a list of candidate genes for future colorectal cancer diagnosis and treatment.

## Background

As the third most common malignancy and the fourth major cause of cancer mortality [1], colorectal cancer is an important threat to human health which accounts for 1 million new cases worldwide each year. The consistency between incidence rates and economic development reflects a westernized lifestyle and attendant risk factor exposures [1]. As a complex condition, colorectal tumor progression is associated with both genetic and environmental factors. To date, only a few common low-penetrance variants attributing to cancer risk have been identified using genome-wide association studies (GWAS), and it is still largely unknown to us the underlying mechanisms and genes involved in tumor development.

Recently, the importance of somatic mutations in cancer development has been widely accepted. It is thought that cancer evolves through the accumulation of somatic mutations in specific genes, depending on various tumor type [2]. Evidence showed that mutation frequency of candidate cancer genes is much higher than expected, and that the particular combination of mutations could influence the tumor's properties [3-6]. These mutations are caused by a combination of environmental and heritable factors [7]. Since the release of the human genome sequence, great efforts have been taken to identify somatic variants in colorectal cancers. For example, Sanger sequencing technique is applied to 13,023 genes and resulted in 189 genes with unexpected excess of somatic mutations in human breast and colorectal cancers [5]. Another group of scientists have used mismatch repair detection (MRD) approach to screen 93 matched tumor-normal sample pairs and 22 cell lines for somatic mutations in 30 cancer relevant genes, and found a total of 152 somatic mutations in breast and colorectal cancers [8], including previously reported genes, such as *BRAF* and *KRAS*.

The recent development of novel high-throughput sequencing methods has provided an unprecedented opportunity to conduct whole-genome scale studies at an affordable cost, and is extensively applied in transcriptome profiling. This method, termed RNA-Seq, gives a far more precise measurement of expression levels of transcripts and a far more sophisticated characterization of their isoforms [9,10], and has brought successes including identification of differentially expressed genes [11], fusion genes in tumor tissue [12-14], allele-specific expressed genes [15,16]. Moreover, it can also serve as an efficient and cost-effective approach to systematically screen variants in transcribed regions [17-20]. To gain insight into the variation spectrum in tumor samples, we developed a sophisticated variant discovery pipeline and applied it to deep-sequencing transcriptome data from 2 colorectal cancer tissues and their matched normal tissues. There are more variants found in tumor tissues than in normal tissues. After additional filters, we also identified tumor-specific mutations in unreported genes, which supplement the increasing list of candidate colorectal cancer genes.

## Methods

### Sequence data

Whole transcriptome sequencing data of paired tumor and normal tissues from 2 stage III colorectal cancer patients were downloaded from NCBI Gene Expression Omnibus (GEO) database (http://www.ncbi.nlm.nih.gov/geo), with the accession number SRP006900. Specifically, 65-bp single-end short reads were generated by Illumina Genome Analyzer, following the standard procedure.

### Sequence alignment

All single-end reads were aligned to UCSC human genome reference assembly (hg19), limited to chromosomes 1–22, X and Y. The alignment was carried out using BWA [21] with default parameters, which allows 4% mismatches in each alignment.

### Variant calling

In each tissue sample, we called variants from the read alignment using SAMtools package [22]. To avoid potential PCR duplicate fragments, we set –D as 100 when invoking vcfutils.pl script, although it varied little when this option is set to 1000 (~3% increase in the total number of variants). Next, we applied several filters to reduce possible false positive calls.

Filter 1.1 We first removed variants that were mistakenly called with a probability greater than 0.01. This was done by requiring a value ≥20 for the ‘QUAL’ column in vcf files generated by SAMtools.

Filter 1.2 We eliminated false positives that were caused by extremely high sequence coverage. To obtain the optimal upper bound for sequence coverage, we searched for variants after filter 1.1 which were also showed in the dbSNP build 135, and assign them as known set. Then, we decided a cut-off value as 97.5% of known variants have lower coverage than that and applied it to the remaining variants. This step was performed independently for each sample.

### Identification of somatic variants

Somatic variants were called by comparing paired normal and tumor tissues. We used custom tools to parse variants after initial filters with following additional filters:

Filter 2.1 Variants in genomic regions of low quality were first excluded for further analysis. Poor quality regions were defined as regions with read coverage in only one sample of a pair, which could be caused by random bias.

Filter 2.2 We next removed variants that were presented in dbSNP135 [23], leaving novel variants.

Filter 2.3 This filter removes variants that are found in both of the matched normal and tumor tissues.

Filter 2.4 To reduce false positives caused by alignment difficulties around indels, we calculated the local mismatch rate as the percentage of mismatches within 10-bp downstream and upstream of a variant. Variants with high local mismatch rate (≥0.1, or ≥2 mismatches) were discarded.

### Gene ontology analysis

The gene ontology (GO) [24] information for genes was assigned using bioconductor (http://www.bioconductor.org) package “org.Hs.eg.db”. The enrichment tests were performed using “topGO” package [25].

## Result

### Read alignment and mutation spectrum

**Figure 1:**
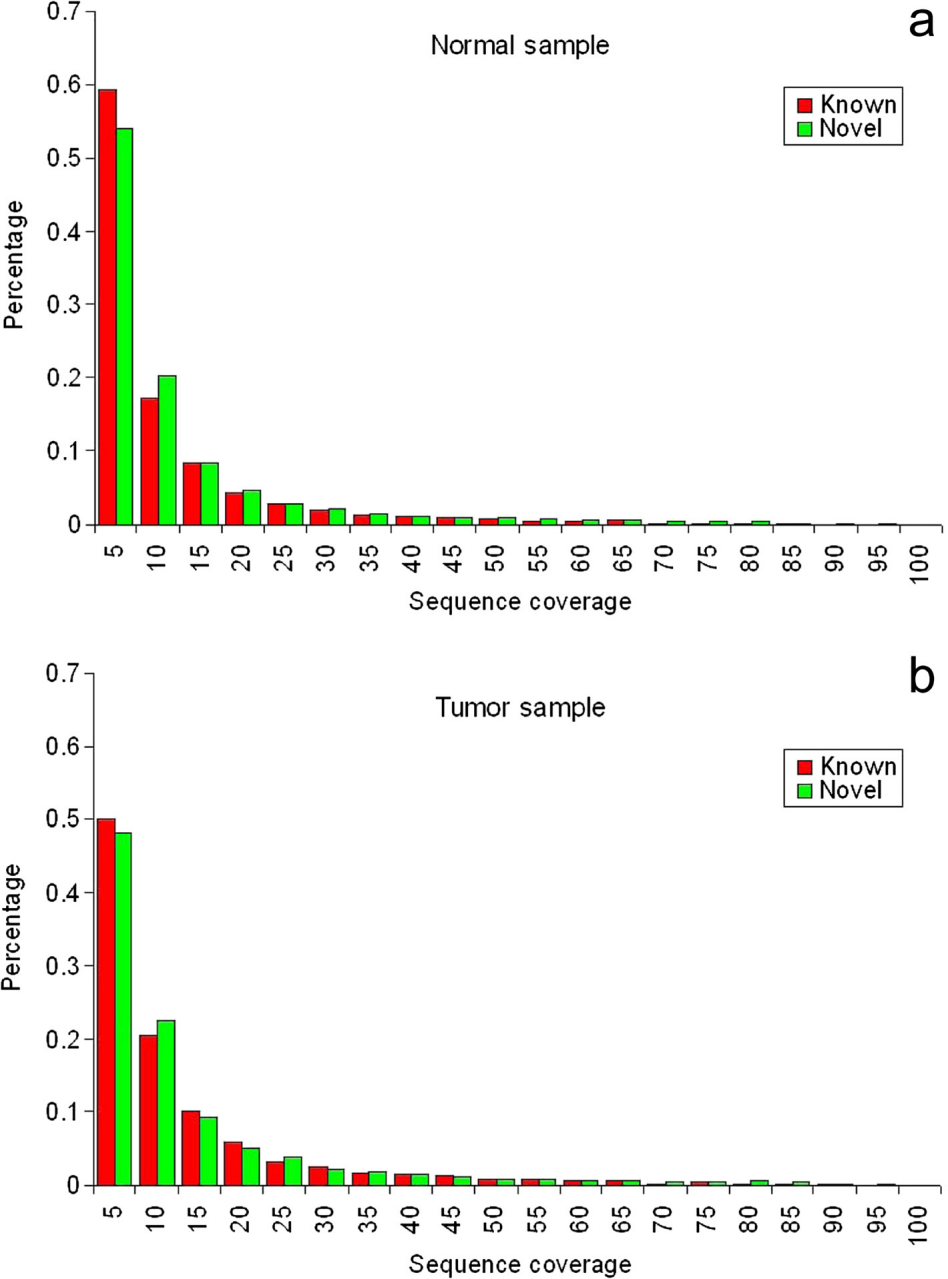
**The distribution of sequence coverage for variant calling.** Known variants are those found in dbSNP 135 database, and novel variants are those identified in this study. **a**. The pattern in normal samples. **b**. The pattern in tumor samples.

**Figure 2:**
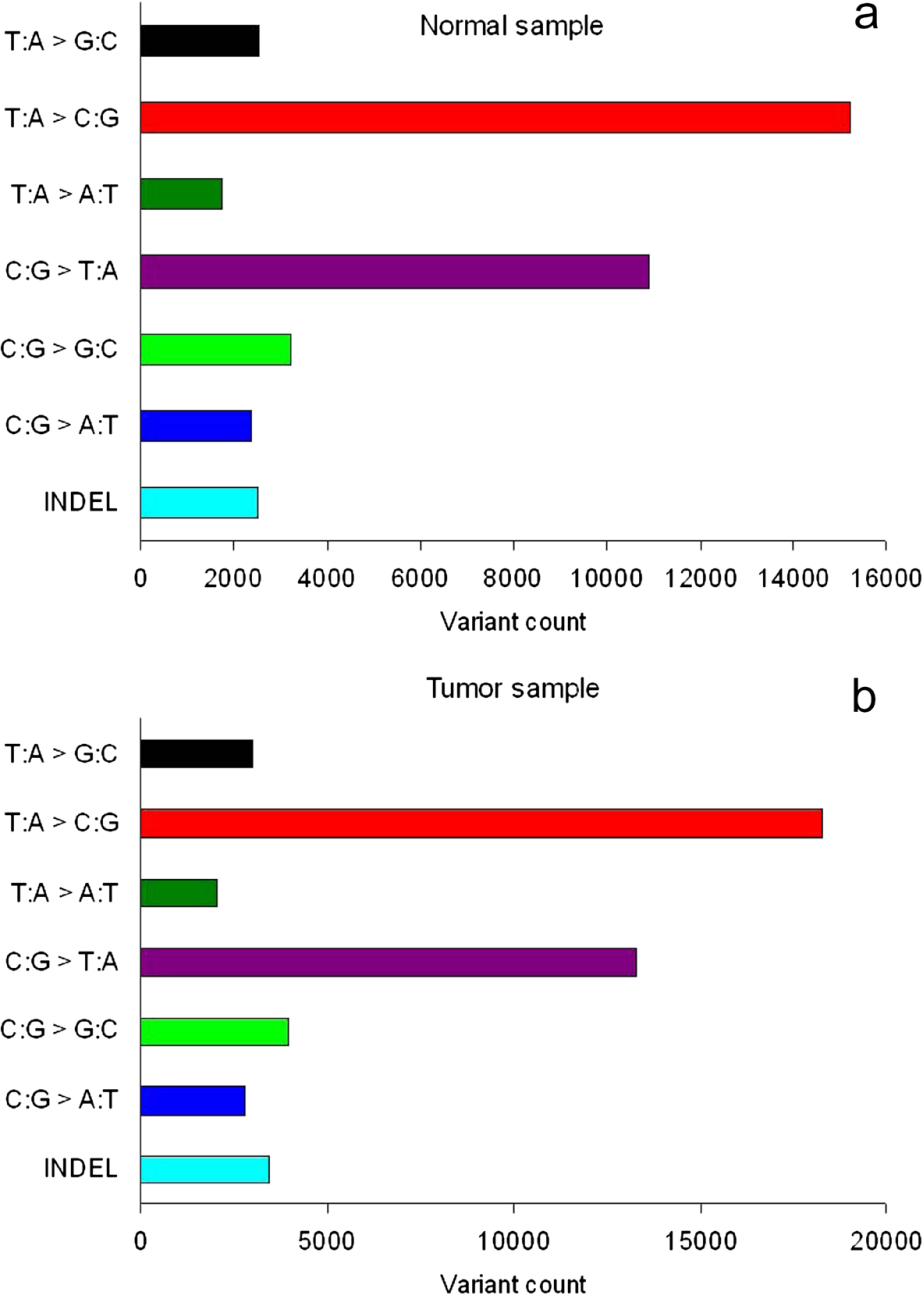
**Mutation spectra of normal and tumor tissues.** The numbers of each of the six classes of base substitution and insertion/deletions are shown. **a**. The pattern in normal samples. **b**. The pattern in tumor samples.

The whole transcriptome data of paired normal and tumor tissues from 2 patients contains ~40 million short reads produced by Illumina Genome Analyzer (9.6 million reads per sample), each 65-bp long. Using BWA aligner [21], we mapped short reads to the human reference genome (hg19), and ~30 million (~76%) short reads were mapped to a unique location (Table 1). Next, we made variant calls using SAMtools package. Since massively parallel sequencing technique has higher error rate, extra care must be taken when we used RNA-Seq to identify variants. Therefore we applied a series of stringent filters to minimize false positive rate. First, we removed variants mistakenly called with a probability greater than 0.01, and obtained 89,129 variants. Since PCR duplicates can cause false positives, we next filtered variants with high sequence coverage. To decide the optimal upper boundary, we denoted known variants as found in dbSNP 135 and novel variants as not, and compared sequence coverage between these two sets. We found that the sequence coverage of known variants is significantly higher than that of novel variants (Figure 1, *P* < 2.2 × 10^-16^, Wilcoxon rank sum test), then we used the 97.5% percentile in known variants (47 reads) as the cutoff to filter potential false positives. After this step, 85,863 variants were remained, and we found that there are more variants in tumor samples when compared to normal samples (23,549 versus 19,383 per sample, ratio = 1.22), with a higher proportion of novel variants in tumor samples (42% versus 39%). Among these variants, a majority are transitions (Figure 2), and the transition/transversion ratio is 2.64 and 2.67 in tumor and normal samples, respectively. These ratios are slightly higher than 2.1, the expected human genome transition/transversion ratio obtained from whole genome resequencing data [26], and it is not unexpected because during transcription, RNA editing specifically changes adenosine (A) to inosine (I), which, in turn, is called as guanosine (G) by sequencers [27].

**Table 1:** Sample and alignment summary

**Sample**	**# reads**	**# unique reads**	**Read length**	**Total throughput**	**Aligned %**
Normal1	9037384	7022993	65 bp	456494545	77.71
Tumor1	8542144	6524738	65 bp	424107970	76.38
Normal2	11308009	8428484	65 bp	547851460	74.54
Tumor2	11461875	8459429	65 bp	549862885	73.80

### Identification of somatic variants

To investigate the potential effect of variants on oncogenesis, we next compared somatic variants between paired normal and tumor samples. Several additional filters were applied to call high confident somatic variants. First, if variant positions were only covered in one sample, we removed them to avoid false positives that are probably caused by sequence bias, resulting in 18,970 and 16,409 tumor and normal variants per sample. Next, we filtered known variants found in dbSNP135 [23], which leads to 11,749 and 9,857 novel variants in each tumor and normal sample, respectively. We also removed variants found in both tumor samples and matched normal samples, as well as variants with a high local mutation rate (2 mismatches in the flanking 20-bp region), which might be a result of local misalignment. In total, we obtained 3,382 tumor-specific novel variants and 1,812 variants per sample, across all autosomes and sex chromosomes.

Of note, the ratio of tumor versus normal samples is significantly higher for novel variants when compared to all variants (3,382/1,812 versus 23,549/19,383, *P* < 2.2 × 10^-16^, Fisher’s exact test), but no bias is observed for transition/transversion ratio between tumor and normal samples (2,054/719 versus 3,929/1,466, *P* = 0.235, Fisher’s exact test), so it is less likely that the excess of somatic variants in tumor samples are due to high false positive rate.

Furthermore, we mapped these somatic variants to protein coding genes to screen for potential important genes for tumor progression. In summary, 1,104 tumor-specific variants and 627 normal-specific variants were found in coding regions. Of them, 671 (60.8%) and 413 (65.9%) variants were disruptive variants (which either change encoding amino acids or reading frames), belonging to 418 and 245 genes, respectively. Additionally, there were 33 genes found to embed somatic mutations in both tumor samples (Table 2).

**Table 2:** List of genes that contain somatic disruptive variants in both tumor samples in this study

**Ensembl ID**	**HNCN symbol**	**Mutation count**
ENSG00000100345	*MYH9*	3
ENSG00000100353	*EIF3D*	2
ENSG00000100461	*RBM23*	2
ENSG00000101182	*PSMA7*	2
ENSG00000108821	*COL1A1*	2
ENSG00000110080	*ST3GAL4*	4
ENSG00000113161	*HMGCR*	2
ENSG00000115457	*IGFBP2*	3
ENSG00000119888	*EPCAM*	3
ENSG00000125124	*BBS2*	2
ENSG00000125970	*RALY*	4
ENSG00000125991	*ERGIC3*	2
ENSG00000128298	*BAIAP2L2*	2
ENSG00000130429	*ARPC1B*	2
ENSG00000134398	*ERN2*	2
ENSG00000144659	*SLC25A38*	2
ENSG00000145113	*MUC4*	10
ENSG00000151846	*PABPC3*	2
ENSG00000163399	*ATP1A1*	2
ENSG00000166794	*PPIB*	2
ENSG00000166888	*STAT6*	3
ENSG00000168542	*COL3A1*	4
ENSG00000173988	*LRRC63*	3
ENSG00000180138	*CSNK1A1L*	2
ENSG00000182944	*EWSR1*	2
ENSG00000184840	*TMED9*	3
ENSG00000188846	*RPL14*	2
ENSG00000197324	*LRP10*	2
ENSG00000198788	*MUC2*	2
ENSG00000204628	*GNB2L1*	4
ENSG00000205277	*MUC12*	2
ENSG00000215570	―	4

### Functional characterization of genes with somatic variants

It is of great interest to understand functions and putative contributions of genes bearing tumor- and normal-specific variants, thus we extracted gene ontology (GO) [24] annotation for these genes and performed gene enrichment analysis. 5 biological processes were found enriched in tumor samples (Table 3), compared to none in matched normal samples. Among these processes is protein localization (GO:0008104) related to tumor development. Researches found that aberrantly localized proteins have been linked to human diseases, including cancers [28-30], suggesting that variants we identified here may promote tumor progression through this process. We also found that tumor-specific variants were enriched in several molecular functions including nucleotide binding (GO: 0000166), which is not unexpected, as several nucleotide binding genes, such as *GNB2L1*, are found to be involved in cancers [31].

**Table 3:** Enriched molecular function categories in GO analysis

**GO.ID**	**Term**	**Annotated**	**Significant**	**Expected**	***P*-value**	**Corrected *P***
*Biological process*						
GO:0044419	interspecies interaction between organisms	397	28	9.22	1.80E-07	0.001729
GO:0033036	macromolecule localization	1443	61	33.5	2.70E-06	0.010247
GO:0051704	multi-organism process	943	45	21.89	3.20E-06	0.010247
GO:0008104	protein localization	1190	52	27.63	6.60E-06	0.015852
GO:0030030	cell projection organization	712	35	16.53	2.30E-05	0.044192
*Molecular function*						
GO:0005515	protein binding	7367	235	171.36	6.30E-12	2.26E-08
GO:0000166	nucleotide binding	2307	84	53.66	1.30E-05	0.01791
GO:0005488	binding	12172	314	283.12	1.50E-05	0.01791
GO.ID	Term	Annotated	Significant	Expected	*P*-value	Corrected *P*
*Biological process*						
GO:0044419	interspecies interaction between organisms	397	28	9.22	1.80E-07	0.001729
GO:0033036	macromolecule localization	1443	61	33.5	2.70E-06	0.010247
GO:0051704	multi-organism process	943	45	21.89	3.20E-06	0.010247
GO:0008104	protein localization	1190	52	27.63	6.60E-06	0.015852
GO:0030030	cell projection organization	712	35	16.53	2.30E-05	0.044192
*Molecular function*						
GO:0005515	protein binding	7367	235	171.36	6.30E-12	2.26E-08
GO:0000166	nucleotide binding	2307	84	53.66	1.30E-05	0.01791
GO:0005488	binding	12172	314	283.12	1.50E-05	0.01791

### Characterization of potential colorectal cancer genes

As is well-known, accumulation of somatic variants is the basic mechanism leading to the development of malignancy. Due to the development of massively parallel sequencing, which makes large-scale sequencing affordable and available, we witnessed a rapid accumulation of somatic variants found in colorectal cancer, such as *MLH3, BRAF, GALNT12,* and *TP53*[32-36]. In the present analysis, we have identified 418 genes with somatic disruptive variants in two tumor samples. Among these genes, we found previously identified genes, such as *TP53*, and tumor-related or oncogenes, such as *RAB5C*, *PIM-3*, *TPT1*, *ST14*. Here we only present several high confident candidate genes that were found in both tumor samples and were good target for diagnosis marker and drug development. Guanine nucleotide binding protein (G protein), beta polypeptide 2-like 1 (*GNB2L1)*, which is also known as *RACK1*, encodes a ubiquitously expressed scaffolding protein and plays a crucial regulatory role in tumor growth [37]. We have detected a 1-bp insertion in both tumor samples, and another 2-bp insertion and a C->T point mutation in one tumor sample. These changes could impact the normal function of *GNB2L1* and thus tumor progression. We also found several members of the mucin protein family that have somatic variants in both tumor samples. Mucin proteins are the major constituents of mucus, which is the viscous secretion that covers epithelial surfaces. There were 2 indels in *MUC2*, 10 indels and point variants in *MUC4*, as well as 1 indel and 1 point variant in *MUC12*. Since the expression of mucin proteins has been correlated with aggressiveness of colorectal cancer [38], the excess of disruptive variants in mucin genes further confirmed their importance in colorectal carcinogenesis.

## Discussion

Recent advances in sequencing technologies continuously reduce sequencing costs and increase sequence output at an unprecedented rate, making RNA-Seq an appropriate method to characterize transcriptome profiles, such as gene expression differences or splicing variations. Wang et al. also used RNA-Seq data to derive sample-specific protein databases [39]. By applying this method to two colorectal cancer cell lines SW480 and RKO, they found a significant improvement in protein identification. In addition, RNA-Seq can also be used for variant detection in transcribed regions, which is suitable for identification of somatic mutations [17-20,40,41]. However, it has been concerned that variant-calling by RNA-Seq is prone to error [18] and could generate a high false discovery rate. To minimize that, we implemented a series of stringent filters in our bioinformatic discovery pipeline. First, we required each variant should have a quality score no less than 20, removing poorly called variants. Next, we used variants that were found in dbSNP135 dataset to train our pipeline and filtered variants with extremely high read coverage. We also applied additional stringent filters to call high confident tissue-specific novel variants, including removing variants with high local mismatch rate. In our final list, we identified more somatic variants in tumor samples than in normal samples, and some variants were in tumor-related genes. Due to our strict filters, we argued that there should be more genes containing tumor-specific somatic variants.

It is widely acknowledged that accumulations of mutations in oncogenes and tumor suppressor genes are the main cause of human cancer [2]. Mutations occurred only in tumor tissues provide important information to understand the potential biological processes underlying carcinogenesis, as well as to facilitate the development of diagnostic and therapeutic markers. As the development of sequencing techniques and the decrease of corresponding costs, large-scale studies begin to accumulate to identify somatic mutations in colorectal cancers. In one study, Sjöblom et al. used polymerase chain reaction (PCR) approach to analyze 13,023 genes in 11 breast and 11 colorectal cancers [5], and found an average of ~90 mutated genes per tumor sample. Using stringent criteria, they identified 189 significantly mutated genes, which affect a wide range of cellular functions, including transcription, adhesion, and invasion. In another study, Timmerman et al. applied next-generation sequencing to sequence the whole exome of primary colon tumors as well as adjacent not affected normal colonic tissue [32]. More than 50,000 small nucleotide variations were identified for each tissue, and there are 359 and 45 most significant mutations in microsatellite stable (MSS) and microsatellite instable (MSI) colon cancers. Somatic mutations were found in the intracellular kinase domain of bone morphogenetic protein receptor 1A, *BMPR1A*, of which germline mutations are associated with juvenile polyposis syndrome. In this present study, we analyzed RNA-Seq data from 2 colorectal tumors and their matched normal tissues to compare their mutation spectra. In general, tumor tissues were enriched in somatic variants compared with normal tissues. By mapping short reads to 54,665 annotated human genes, we have detected 418 genes with somatic variants in tumor tissues, including 3 mucin genes found in both tumor samples. Mucins are complex glycoproteins and play important roles in protecting epithelial surfaces [38], alterations in mucin expression and the extent of their glycosylation have been reported to be associated with neoplastic progression and metastasis in several human cancers [42-44]. Since disruptive variants may radically change protein functions instead of gene expression, we further used SIFT tool [45] to assess their effects on protein functions. 10 of 12 variants were classified as tolerated variants, which have a limited impact on the protein function. Thus it is more likely that these disruptive mutations in mucin genes regulate gene expression and thus lead to tumorigenesis. Additionally, mucins can form insoluble mucous to protect gut lumen, therefore amino acid changes in these genes could result in the modification of the micro-environment. This change may in turn lead to the proliferation of some bacteria such as *Fusobacterium nucleatum* and *Coriobacteria*, which have been reported to be significantly over-represented in colorectal tumor specimens [46,47]. Somatic disruptive mutations in these genes found here suggest the abnormality of their expression is related to colorectal tumorigenesis.

## Conclusions

RNA-Seq is a powerful tool to identify somatic mutations in protein-coding regions after sophisticated filters. The list of genes we found in this study only represents a minimal set of candidate genes, due to the stringent criteria we applied. However, the identification of several oncogenes and tumorigenesis genes, as well as signal pathway genes, provides meaningful candidates to understand the molecular mechanism of colorectal cancer and for future drug target development. Although additional validations and functional examination are helpful, RNA-Seq, with well developed bioinformatic pipeline, can serve as the first step for somatic variant screening in human cancers.

## Competing interests

The authors declare that they have no competing interests.

## Authors' contributions

YZ and SQ carried out the prime studies and drafted the manuscript. All people have participated in the design of the study and the experiments. In addition, YP and LB coordination and helped to draft the manuscript. All authors read and approved the final manuscript.

## Pre-publication history

The pre-publication history for this paper can be accessed here:

http://www.biomedcentral.com/1471-2350/14/32/prepub
